# Unanticipated papillary renal cell carcinoma in a complicated renal cyst: A case of radical nephrectomy following surgical complications

**DOI:** 10.1016/j.eucr.2025.103254

**Published:** 2025-10-21

**Authors:** Anahita Ansari Djafari, Seyyed Ali Hojjati, Azadeh Rakhshan, Tayeb Hosseini, Sina Samenezhad

**Affiliations:** a Urology Department, School of Medicine, Shahid Beheshti University of Medical Sciences, Tehran, Iran; b Laser Application in Medical Sciences Research Center, Shahid Beheshti University of Medical Sciences, Tehran, Iran; c Department of Pathology, Shohada-e-tajrish Educational Hospital, School of Medicine, Shahid Beheshti University of Medical Sciences, Tehran, Iran; d Department of Radiology, Shohada-e-tajrish Hospital, Shahid Beheshti University of Medical Sciences, Tehran, Iran

**Keywords:** Papillary renal cell carcinoma, Cystic renal mass, Bosniak IIF, Radical nephrectomy, Diagnostic challenge, Surgical complication

## Abstract

Cystic renal masses are often presumed benign, especially Bosniak I or IIF lesions. Rarely, papillary renal cell carcinoma (PRCC) can arise within these cysts, posing diagnostic and surgical challenges. We report a 40-year-old male with a right renal cyst under surveillance for three years as Bosniak IIF. Progressive growth led to laparoscopic cyst unroofing, but intraoperative findings of a solid mass and significant bleeding necessitated urgent radical nephrectomy. Histopathology confirmed pT2bN0Mx PRCC with solid and pseudopapillary features. This case underscores imaging limitations, the potential for malignancy in low-risk cysts, and the importance of vigilant monitoring, preoperative planning, and patient counseling.

## Highlights

• **Rare malignant potential:** Papillary renal cell carcinoma can arise within cystic renal lesions initially classified as Bosniak IIF, even after long-term surveillance.
• **Diagnostic limitations:** Imaging, including CT, may not reliably detect malignancy in apparently benign cysts, emphasizing the need for repeat assessment and careful preoperative planning.
• **Surgical challenges:** Unexpected solid components and intraoperative bleeding may necessitate urgent radical nephrectomy, highlighting the importance of patient counseling.
• **Clinical** **implications:** Vigilant follow-up, individualized management, and awareness of both oncologic and perioperative complications are essential to optimize outcomes.

## 1 Introduction

Cystic renal masses constitute a heterogeneous spectrum of lesions with variable malignant potential. Historically, aggressive surgical intervention was often undertaken for indeterminate cystic lesions, reflecting concerns of occult malignancy. This approach has contributed to overtreatment, despite accumulating evidence that the majority of cystic lesions are benign and, when malignant, frequently demonstrate indolent biological behavior compared with solid renal cell carcinomas (RCCs).^1^

The Bosniak classification has long provided a framework for risk stratification of cystic renal masses; however, its earlier versions were limited by ambiguous terminology, incomplete characterization across imaging modalities, and considerable interobserver variability. The 2019 revision of the Bosniak system addressed these limitations by formally integrating MRI, refining definitions of key imaging features, and providing more robust malignancy risk estimates. This update aims to reduce unnecessary surgical interventions while improving diagnostic accuracy and standardization.^1^

Guideline recommendations increasingly align with these refinements. The 2023 Canadian Urological Association (CUA) update endorses Bosniak v2019 for clinical use and specifically recommends active surveillance for Bosniak III and IV lesions ≤2 cm, with surveillance or surgical intervention as reasonable alternatives for 2–4 cm lesions. These decisions should be individualized through shared decision-making, acknowledging the limited certainty of available evidence.^2^

Nevertheless, even lesions originally classified as Bosniak I cannot be assumed to remain biologically inert indefinitely. Malignant transformation, although rare, has been documented. A recent case demonstrated progression from a Bosniak I lesion to Bosniak IV over a five-year interval, ultimately confirmed as cystic RCC following resection. This underscores the importance of longitudinal imaging when lesion morphology, enhancement, or growth kinetics evolve.^3^

Accurate radiologic characterization remains central to management. The initial diagnostic task is to confirm a lesion's cystic nature, thereby excluding necrotic or lipid-poor solid tumors. Subsequent assessment should employ the Bosniak lexicon across CT, MRI, and, when appropriate, contrast-enhanced ultrasound (CEUS).^4^ CT remains the staging modality of choice, though its limitations in nodal staging are recognized; enhancement patterns may offer superior predictive value for post-ablation viability compared with size criteria alone.^5^ Recent systematic reviews confirm CT as the diagnostic standard, with multiparametric MRI and ultrasound serving as complementary modalities. From a therapeutic standpoint, partial nephrectomy remains the gold standard, while percutaneous ablation is an acceptable option in high-risk patients, and active surveillance is increasingly recognized as appropriate for select cases.^6^ Updated European Association of Urology (EAU) guidelines similarly endorse a personalized, evidence-based approach to localized and advanced RCC, incorporating evolving imaging and treatment paradigms.^7^

In this context, we report a case of a large, presumed simple cyst that demonstrated atypical intraoperative and histopathological features, ultimately diagnosed as papillary RCC. While the occurrence of papillary RCC within cystic lesions is increasingly recognized, this case is notable for the intraoperative challenges and management pitfalls encountered when an ostensibly benign cyst proved malignant. The report underscores the importance of preoperative contingency planning, ongoing vigilance in surgical decision-making, and comprehensive patient counseling.

## 2 Case report

A 40-year-old male presented to our hospital with complaints of left-sided flank pain. His medical history was unremarkable, and he had no significant past medical conditions. The patient was a heavy smoker, had no history of alcohol use, and was not on any medications. He had a BMI of 30. His primary concern was an obstructing stone in his left kidney(Fig. 1-A).

**Fig. 1-A:**
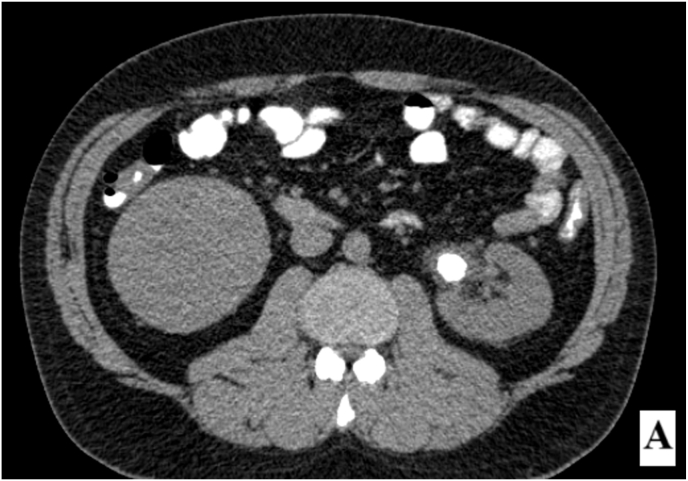
Stone in Renal Pelvis of Left kidney.

The patient also had a history of a large, asymptomatic cyst located in the lower pole of his right kidney, which had been under surveillance via ultrasound for three years. The most recent ultrasound demonstrated a rounded, homogeneous, anechoic mass with a thin wall and no calcification. Over three years, the cyst had increased in size from 70 × 55 mm to 110 x 90 mm. The cyst was classified as Bosniak IIf, with no enhancement on imaging. Given its stable and benign features, the cyst was not considered a surgical candidate, and surveillance continued.

Given the progressive growth of the cyst, a more detailed abdominal pelvic CT scan with IV contrast and delayed phase was ordered to assess both the obstructing stone in the left kidney and the right renal cyst. The CT scan revealed a 13 × 21 mm stone in the left kidney, which was obstructing the renal pelvis. Additionally, the CT identified a large, well-defined, poorly enhancing cyst in the lower pole of the right kidney, measuring 110 × 90 mm. The lesion demonstrated attenuation values within the hypoenhancing range (<30 HU post-contrast), consistent with a low-attenuation lesion showing minimal contrast uptake (Fig. 1-B).

**Fig. 1-B:**
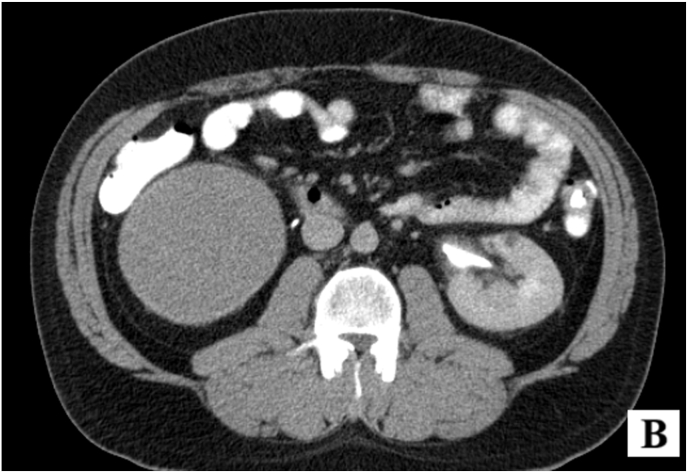
Poorly enhancing cyst in the lower pole of the right kidney.

The patient was then scheduled for left-sided percutaneous nephrolithotomy (PCNL) for the obstructing renal stone. The procedure was successfully performed without complications, and the stone was removed. Following the procedure, it was agreed that the right renal cyst would be monitored with ultrasound every six months, given its stable and asymptomatic nature.

Six months later, the patient returned with mild left-sided flank pain and expressed a desire for definitive treatment of the right-sided cyst. No recent imaging had been performed, In hindsight, repeat cross-sectional imaging before surgical intervention would have been advisable to reassess morphology and enhancement characteristics. The decision was made to proceed with laparoscopic unroofing of the cyst, which was discussed with the patient, and consent was obtained. Preoperative evaluation showed the patient to be hemodynamically stable, with a blood pressure of 120/80 mmHg, a heart rate of 74 bpm, and a hemoglobin level of 13 g/dL. All other laboratory tests were within normal limits.

During surgery, a diamond-shaped trocar was placed, and the colon was medialized to allow access to Gerota's fascia. Upon exploration, a tense and large cyst was encountered, and there was no clear demarcation between the cyst and the renal cortex. Given the unexpected findings, the cyst was aspirated with a fine needle, but this was unsuccessful, and the mass appeared to be solid. Given this unexpected finding, the operative plan was reassessed. Because all preoperative imaging had suggested a benign cyst and informed consent for nephrectomy had not been obtained, a limited core needle biopsy was performed to obtain histologic confirmation rather than proceeding with definitive resection. Intraoperative frozen-section analysis was unavailable at our institution, and immediate nephrectomy without consent or histologic evidence of malignancy was deemed inappropriate. This conservative approach aimed to balance patient safety with ethical considerations. A core needle biopsy was performed on different sections of the mass. Biopsy resulted in minimal bleeding, which was effectively managed using hemostatic agents (Surgicel and cautery).

Approximately 2 h post-surgery, while in the recovery room, the patient developed tachycardia and hypotension. His blood pressure dropped to 100/70 mmHg, and his drain had accumulated approximately 600 mL of blood. After stabilization, an interventional radiology consultation was requested. Angiography of the right renal artery revealed a large mass in the lower pole of the right kidney, but no active arterial bleeding was identified. Angiographies of the superior mesenteric artery (SMA), celiac artery, and lumbar veins were also conducted, with no evidence of active arterial extravasation (Fig. 1-C).

**Fig. 1-C:**
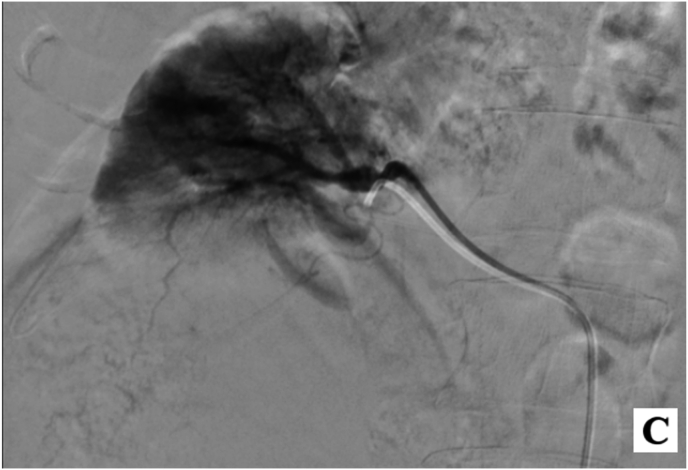
Angiography of the right renal artery.

Despite initial stabilization, the patient's condition continued to deteriorate with worsening hypotension (80/60 mmHg), tachycardia (HR 110 bpm), and a hemoglobin level dropping to 9 g/dL. Given the clinical deterioration, the decision was made for urgent open laparotomy. The patient was transferred to the operating room, where a collaborative approach between the urology and surgical teams was employed.

Intraoperatively, no injury to the hepatic, splenic, or intestinal structures was noted. However, oozing from the renal mass was observed, prompting the decision for radical nephrectomy. The entire right kidney was excised (Fig. 2-A), and the patient was transfused with two units of packed red blood cells. Postoperatively, the patient was stable and transferred to the ward.

**Fig. 2-A:**
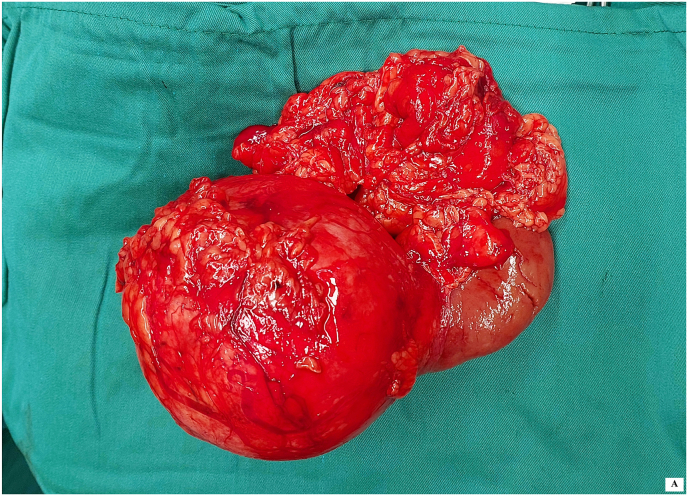
Excised right kidney.

Two days after surgery, the patient developed tachycardia and a decrease in oxygen saturation. A CT pulmonary angiogram revealed a subsegmental pulmonary embolism (PTE), which was managed with appropriate anticoagulation. Despite this complication, the patient made a full recovery and was discharged on the tenth day following surgery.

Pathology of the excised kidney revealed a 10 x 6 × 4 cm exophytic mass, located in the lower pole, without invasion into the perirenal fat or renal sinus (Fig. 2-B). Microscopic examination demonstrated a neoplasm with compressed tubular and papillary structures, forming a pseudopapillary pattern. The tumor cells had low-grade nuclei, eosinophilic to clear cytoplasm, and aggregates of foamy macrophages. Mitotic activity was low, and necrosis was absent (Fig. 3). Immunohistochemistry revealed cytoplasmic staining for CK7 and AMACR in the neoplastic cells, with negative staining in the foamy histiocytes. The final diagnosis was papillary renal cell carcinoma (PRCC) with solid and pseudopapillary patterns (Fig. 4-A, Fig. 4-B-A and 4-B). The tumor was staged as pT2bN0Mx, with all surgical margins free of tumor.

**Fig. 2-B:**
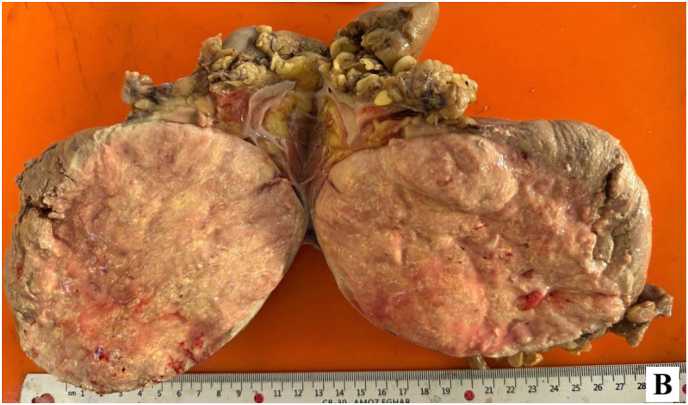
Macroscopic examination shows kidney (14x10 × 6cm) with an exophytic well circumscribed mass (10x6x4cm).

**Fig. 3:**
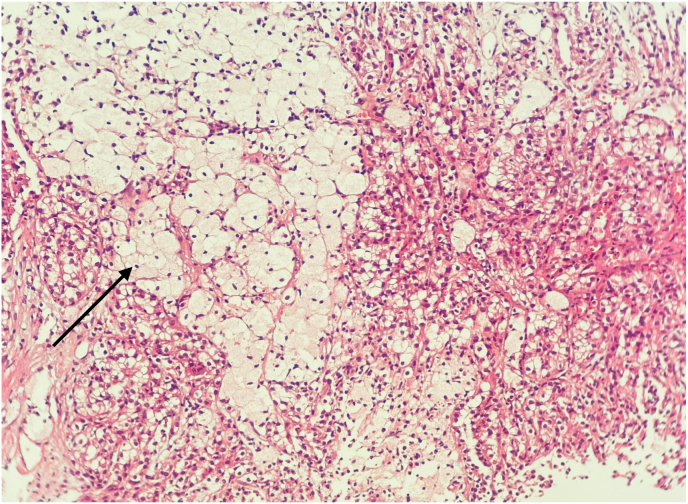
Microscopic view of Mass.

**Fig. 4-A:**
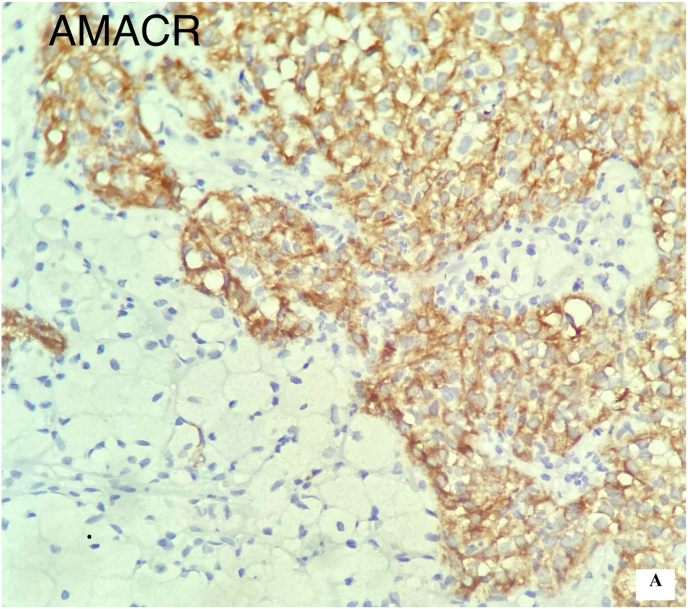
Immunostaining with CK7.

**Fig. 4-B:**
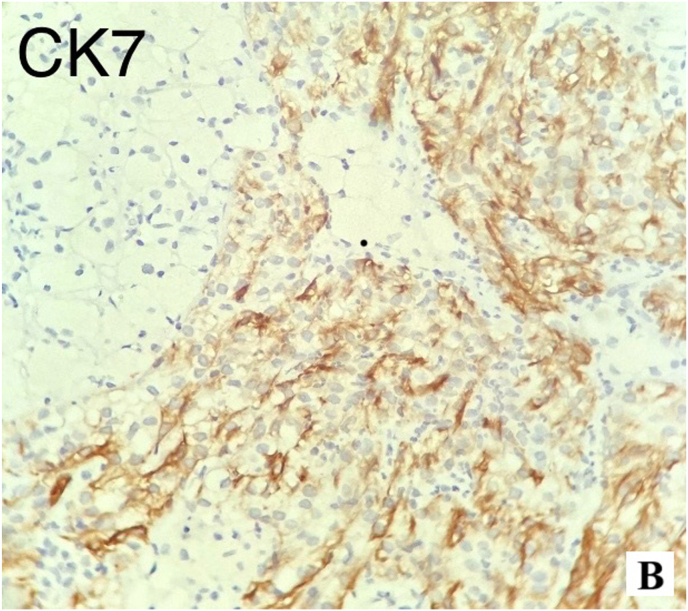
Immunostaining with AMACR

The patient's follow-up CT scan with IV contrast, performed six months post-surgery, showed no recurrence of the tumor.

## 3 Discussion

Cystic renal masses are often assumed to be benign, and the Bosniak classification has served as the cornerstone for risk stratification for more than three decades. Nonetheless, papillary renal cell carcinoma (PRCC) arising within lesions radiologically classified as benign is increasingly recognized as a diagnostic pitfall. Rana et al. reported a Bosniak II cyst that was ultimately confirmed to be PRCC on histology, underlining that even lesions categorized as low risk may conceal malignant pathology.^8^ Other case reports similarly describe RCC developing in presumed simple or minimally complex cysts, reinforcing that reliance solely on imaging may underestimate oncologic risk.^9^

Our case aligns with prior descriptions in which papillary RCC masqueraded as a benign cyst, phenomenon explained by the well-documented hypoenhancing nature of papillary tumors on contrast-enhanced CT^10^ However, it diverges in several respects. Most published cases involve relatively small lesions that were resected uneventfully,^8^^,^^9^ whereas our patient presented with a larger cystic mass complicated by intraoperative bleeding. Discrepancies may be explained by radiologic limitations: papillary tumors often demonstrate low attenuation and homogeneous appearance, which can mimic simple cysts on CT,^11^ Postoperative materials can also mimic renal tumors on imaging; retained oxidized cellulose (Surgicel®) has been reported to appear as an enhancing mass resembling recurrence, emphasizing awareness of this pitfall to avoid unnecessary surgery.^12^^,^^13^

Quantitative CT evaluation, including Hounsfield unit (HU) measurements, can aid in differentiating cystic from solid or infected renal lesions. Recent prospective studies have confirmed the diagnostic reliability of HU-based analysis in urologic imaging, underscoring its role in distinguishing clinically similar entities such as hydronephrosis and pyonephrosis.^14^ Growth kinetics may also play a role, as papillary RCC can remain indolent before rapid progression. Furthermore, percutaneous biopsy proposed by some as an adjunct for Bosniak IIF lesions was not performed, which might otherwise have raised suspicion of malignancy earlier.^15^

Beyond the diagnostic aspect, this case underscores the importance of surgical preparedness and intraoperative adaptability when managing lesions presumed benign. As our experience demonstrated, encountering unexpected solid components or hemorrhage during presumed cyst unroofing necessitates an immediate shift in strategy. Surgeons should maintain a predefined contingency plan, including readiness for conversion to open surgery or radical nephrectomy if malignancy or uncontrolled bleeding is encountered. A recent case of renal schwannoma misdiagnosed as angiomyolipoma similarly highlighted how preoperative misclassification and limited biopsy accuracy can mislead surgical planning, emphasizing the need for multidisciplinary review and flexibility during intervention.^16^

These findings have important implications for managing Bosniak IIF cysts. Although the Bosniak 2019 revision provides greater clarity in lesion characterization, atypical papillary tumors may still escape detection within the benign spectrum.^17^ Recent reports have extended active surveillance to Bosniak III and select IV lesions with encouraging short-term safety.^18^ Yet, our case highlights the residual risk that even IIF lesions may harbor carcinoma, underscoring the need for detailed counseling regarding both oncologic uncertainty and operative morbidity. Prognostic series indicate that cystic RCCs, including papillary variants, generally demonstrate more favorable survival compared with conventional clear cell RCC,^19^ but this potential advantage must be weighed against risks of surgical complications such as those encountered here.

The primary limitation of our report is its single-case nature, which restricts generalizability. While the case aligns with prior literature suggesting that malignant transformation within presumed benign cysts is rare but possible, definitive conclusions cannot be drawn. Additionally, our follow-up duration is limited, precluding assessment of long-term oncologic outcomes.

## 4 Conclusion

This case illustrates the diagnostic challenge of papillary RCC arising within a Bosniak IIf cyst, where its well-known hypoenhancing behavior can mimic benignity and lead to underestimation of oncologic risk. It emphasizes that radiologic reassurance does not fully exclude malignancy, and both unexpected oncologic findings and operative complications must be considered in counseling and surgical planning. Until stronger evidence guides management, vigilant follow-up and thorough informed consent remain essential.

## CRediT authorship contribution statement

**Anahita Ansari Djafari:** Writing – review & editing, Supervision, Project administration, Conceptualization. **Seyyed Ali Hojjati:** Validation, Methodology, Data curation, Conceptualization. **Azadeh Rakhshan:** Visualization, Resources, Formal analysis, Data curation. **Tayeb Hosseini:** Visualization, Validation. **Sina Samenezhad:** Writing – review & editing, Writing – original draft, Visualization, Validation, Project administration, Methodology, Investigation, Data curation, Conceptualization.

## Patient consent

Patient informed consent was obtained to publish her information. The patient's private information remained confidential with the researchers.

## Ethical approval

This study was reviewed by our hospital Institutional Review Board (IRB) and was deemed exempt from formal ethical approval because the treatment and data collection were based entirely on established clinical guidelines and standard urological practice performed by an expert urologist. No experimental interventions or novel protocols were involved, and all actions followed evidence-based, routine medical care.

## Funding

This research received no specific grant from any funding agency in the public, commercial, or not-for-profit sectors.

## Declaration of competing interest

The authors declare no competing financial or personal interests.
